# Novel Pathway for Efficient Covalent Modification of Polyester Materials of Different Design to Prepare Biomimetic Surfaces

**DOI:** 10.3390/polym10121299

**Published:** 2018-11-23

**Authors:** Viktor Korzhikov-Vlakh, Ilia Averianov, Ekaterina Sinitsyna, Yuliya Nashchekina, Dmitry Polyakov, Ivan Guryanov, Antonina Lavrentieva, Lukas Raddatz, Evgenia Korzhikova-Vlakh, Thomas Scheper, Tatiana Tennikova

**Affiliations:** 1 Institute of Chemistry, Saint-Petersburg State University, St. Petersburg 199034, Russia; v_korzhikov@mail.ru (V.K.-V.); ivan.guryanov1@gmail.com (I.G.); 2 Institute of Macromolecular Compounds, Russian Academy of Sciences, St. Petersburg 199004, Russia; averianovilia@gmail.com (I.A.); kat_sinitsyna@mail.ru (E.S.); vlakh@mail.ru (E.K.-V.); 3 Institute of Cytology, Russian Academy of Sciences, St. Petersburg 194064, Russia; ulychka@mail.ru; 4 Institute of Experimental Medicine, Russian Academy of Sciences, St. Petersburg 197376, Russia; ravendoctor@mail.ru; 5 Institute of Technical Chemistry, Gottfried-Wilhelm-Leibniz University of Hannover, 30167 Hannover, Germany; lavrentieva@iftc.uni-hannover.de (A.L.); raddatz@iftc.uni-hannover.de (L.R.); scheper@iftc.uni-hannover.de (T.S.)

**Keywords:** biofunctionalization, polyvinylsaccharide, polyesters, PLA, PCL, bioligands, scaffolds for bone tissue engineering, nanoparticles for drug delivery, cells adhesion, phagocytosis

## Abstract

To form modern materials with biomimic surfaces, the novel pathway for surface functionalization with specific ligands of well-known and widely used polyester-based rigid media was developed and optimized. Two types of material bases, namely, poly(lactic acid) and poly(ε-caprolactone), as well as two types of material design, e.g., supermacroporous matrices and nanoparticles (NPs), were modified via covalent attachment of preliminary oxidized polyvinylsaccharide poly(2-deoxy-*N*-methacryloylamido-d-glucose) (PMAG). This polymer, being highly biocompatible and bioinspired, was used to enhance hydrophilicity of the polymer surface and to provide the elevated concentration of reactive groups required for covalent binding of bioligands of choice. The specialties of the interaction of PMAG and its preliminary formed bioconjugates with a chemically activated polyester surface were studied and thoroughly discussed. The supermacroporous materials modified with cell adhesion motifs and Arg-Gly-Asp-containing peptide (RGD-peptide) were tested in the experiments on bone tissue engineering. In turn, the NPs were modified with bioligands (“self-peptide” or camel antibodies) to control their phagocytosis that can be important, for example, for the preparation of drug delivery systems.

## 1. Introduction

Polyester-based materials represent an important field of research due to their prospective and widely used applications in biomedicine [1,2,3]. Among these, the formation of the supermacroporous matrices to be used as the scaffolds for tissue engineering [4], as well as the particulate formulations with encapsulated drugs [5,6] have attracted the most interest. The formation of such materials based on poly(lactic acid) (PLA) and poly(ε-caprolactone) (PCL) is not only a scientific challenge, but also represents significant commercial interest because of their biocompatibility/biodegradability and thus they were approved by the Food and Drug Administration (FDA) for medical applications [7,8].

However, according to the description of biomaterials evolution given by Langer [9] and other researchers [10,11,12,13,14], PLA and PCL alone represent only the biomaterials of the third generation. A future, fourth generation should be biomimetic [10,15,16,17], e.g., they have to interact with cells and tissues in a biospecific manner and resemble the composition and properties of natural tissues. As for the materials intended for scaffold preparation, they have to mimic the extracellular matrix of the tissue to be regenerated and to provide the appropriate cell-instructive signals for cells to adhere, migrate, grow and differentiate [16,18]. In the case of drug delivery formulations, the surface composition of the particles has to allow for the control over their early elimination from the blood by phagocytosis, as well as to actively target them to some tissue-specific cellular receptors [19,20].

In order to provide the materials with the abovementioned properties, the surface biofunctionalization is required [16,21]. Such biofunctionalization is implied as attachment of biologically active ligands into the material structure in order to govern its biospecific interaction with cells. The choice between non-covalent and covalent attachment has to be done at this point [22]. The non-covalent binding is easy to perform, but in the most cases, this way is not suitable because it does not provide appropriate stability of the bond between surface and biomolecule with such aggressive and multicomponent media as biological fluids. It could be a serious drawback, especially in the case of adhesion motifs, such as polycations and RGD-peptides, which are quite toxic in the solution [23,24].

The covalent attachment of bioligands to the surface generally provides more control and stability. The most serious drawback of this type of immobilization of biomolecules is the partial loss of their activity. To minimize this peculiarity, the attachment of bioligands via a spacer of different nature and length is generally performed [25,26]. The spacer provides the steric accessibility of bioligands, and prevents the conformation changes associated with biomolecule-surface interactions. The covalent binding of biomolecules also requires tailored chemistry of attachment. The reactions have to be fast, unambiguous, safe for biomolecules, easy-to-perform, and the rest of the functional groups should be easily inactivated to exclude unsuspected reactions [26].

When dealing with polyesters’ biofunctionalization, one will face their relative hydrophobicity and lack of reactive groups for biomolecule attachment [27,28,29]. Only the terminal hydroxyl and carboxylic groups are available for modification without chain cleavage. Many efforts have been performed to modify the surface of the polyester-based materials. In particular, lots of studies have utilized PLA-PEG block-copolymers [30]. Application of PEG with terminal amino [31] or maleimide [32] groups allowed for introduction of appropriate chemistry for bioligands attachment. Another useful approach for polyester surface modification is special treatment of the surface to generate the functional groups for direct bioligands attachment. Such methods include the treatment with plasma [33,34], sodium hydroxide [35] and enzymatically catalyzed hydrolysis [36,37].

The promising results on modification of polyesters’ surfaces with natural polysaccharides were shown in some papers [38,39,40,41]. This approach allowed for making the surface of materials more attractive for the cells and provided specific cells-biomaterial interactions via attachment of adhesion motifs and growth factors [42]. Nevertheless, natural polysaccharides could undergo fast degradation leading to detachment of bound bioligands.

In our previous studies we suggested the application of the oxidized poly(2-deoxy-*N*-methacryloylamido-d-glucose) (ox-PMAG) with reactive aldehyde groups to functionalize the bioceramic (Sponceram^®^) surface [43,44]. The results obtained clearly showed that the application of polyvinylsaccharides allowed for controllable attachment of several bioligands, which provided the synergetic effect and induced cell adhesion, differentiation and tissue formation [45]. In this case, the chosen macromolecular spacer, being highly biocompatible and biosimilar, as well bearing the active bioligands, played the role of some vector defining cells behavior. 

In this paper, we suggested the pathway based on application of ox-PMAG to construct the differently designed biomimetic materials based on PLA and PCL, namely, supermacroporous matrices (formed by thermally induced phase separation) and nanoparticles (obtained using the single emulsion approach). The strategy and benefits of the modification of polyesters with polyvinylsaccharides is outlined. In the case of scaffolds formation, the effect of biofunctionalization on mesenchymal stem cell adhesion and differentiation was tested. Biofunctionalization of PLA-based particles was done to control their phagocytosis by primary peritoneal macrophages.

## 2. Materials and Methods 

### 2.1. Materials 

PLA (SEC: *M_w_* = 150,000; *M_n_* = 95,540; Ð = 1.57) and PCL (SEC: *M_w_* = 117,000; *M_n_* = 81,250; Ð = 1.44) were obtained by stannous octoate initiated ring-opening polymerization (ROP) of d,l-lactide and ε-caprolactone (Sigma-Aldrich, Darmstadt, Germany) according to previously described procedures [46,47]. Oxidized poly(2-deoxy-*N*-methacriloylamido-d-glucose) (ox-PMAG; *M_w_* (SEC) = 18,000, Ð = 2.21; [CHO] = 35 ± 7 mol%) was synthesized via free-radical polymerization of *N*-methacryloylamido-d-glucose (MAG, obtained from glucosamine, Aldrich, Munich, Germany, as described in [43]), followed by oxidation of the polymer obtained using the reaction with sodium periodate, as published earlier [43]. All reagents for polymer modification: Sodium periodate, sodium hydroxide, 1,2-ethylenediamine (EDA), 1-Ethyl-3-(3-dimethylaminopropyl)carbodiimide (EDC), 1-hydroxybezotriazole (HOBt), bovine serum albumin (BSA) and chymotrypsinogen (CTRG), glycine (Gly) etc.; all analytical reagents, such as Schiff’s reagent and 2,4,6-trinitrobenzene sulfonic acid (TNBS), as well as buffer salts were purchased from Fluka (Buchs, Switzerland) and Sigma (Darmstadt, Germany) and used without additional purification. 1,4-dioxane, THF, methanol and chloroform of HPLC grade were purchased from Merck (Darmstadt, Germany). 

GRGDSP (RGD-peptide) and GNYTCEVTELTREGETIIELK (“self-peptide” [48]) were synthesized via conventional solid-phase peptide synthesis using Fmoc-strategy. Camel polyclonal antibodies were kindly donated by Dr. L. Churilov (Medicinal Faculty, Saint-Petersburg State University). Fluorescent labels Cy3-NHS and 4′,6-diamidino-2-phenylindole (DAPI) were purchased from Lumiprobe (Russia, Moscow). Vivaspin-columns (Sartorius Group, Göttingen, Germany) with membrane of MWCO 3000, 10,000 and 30,000 were used for ultrafiltration in centrifuge during polymer bioconjugates and particles purification.

### 2.2. Instruments

The magnetic stirrer MR Hei-Mix S (Heidolph, Schwabach, Germany), Schlenk reaction tubes with rubber septum (Aldrich, Munich, Germany) and rotary evaporator Hei-VAP Precision ML/G3B (Schwabach, Heidolph, Germany) were used for polymer synthesis. The latter was also applied for oil phase evaporation at particle preparation. LC-10 Shimadzu system supplied with refractometric detector and Waters Styragel HMW 6E analytical column were applied for size-exclusion chromatography (SEC) of polymers. The LT-912 LOIP (St. Petersburg, Russia) cryothermostat was used for controllable cryogelation of polymer solutions at supermacroporous matrices preparation. FreeZone 1L freeze dryer (Labconco, Kanzas City, MO, USA) was used for drying of the samples. The ultrasound homogenizer Sonopuls HD2070 Bandelin (Berlin, Germany) was used for emulsification during particle preparation. The TS-100C thermo-shaker BioSan (Riga, Latvia) was applied for stirring and thermocontrol over polymer and particle modification reactions. The Vivaspin-column dialysis and particle separation were performed with Sigma 2–16 KL centrifuge (Sigma, Darmstadt, Germany). The modification of polyester-based porous matrices was conducted at the Unimax 1010 rotation shaker and incubator (Heidolph, Schwabach, Germany). For UV–VIS measurements the UV 1800 Shimadzu spectrophotometer (Kyoto, Japan) and NanoDrop ND-2000C (Thermo Fisher Scientific, Waltham, MA, USA) were used.

The morphology of samples obtained was imaged using scanning electron microscope (SEM) Zeiss Supra 55VP (Oberkochen, Germany). The mean size of lyophilized particles and pore size were estimated from SEM images using free ImageJ software. For measurement of particle size distribution, the dynamic light scattering (DLS) Zetasizer Nano ZS (Malvern, Enigma Business Park, UK) was used. The optical microscope Nikon Eclipse E600FN (Tokyo, Japan) and laser confocal microscope LSM 510 Meta (Carl Zeiss, Basel, Switzerland) were used for cell imaging. Microplate Spectrophotometer-Fluorometer Fluoroskan Ascent reader (Thermo Fisher Scientific, Waltham, MA, USA) was used for quantitative measurements in cell culture experiments.

### 2.3. Chemical Methods

#### 2.3.1. Materials Preparation 

##### Supermacroporous Matrices

The formation of PLA- and PCL-based porous materials was performed via the previously published procedure [46]. In brief, the polymer was dissolved in a dioxane-water mixture. The formed solution was kept at 60 °С for 30 min and then cooled in a cryothermostat to the temperature corresponding to the metastable state [46] of the system at a constant rate of 2 K/min. The solution was kept at that temperature for 2 min and then was placed for several minutes in liquid nitrogen. The sample was then freeze-dried. 

##### Nanoparticles

The PLA- and PCL-based nanoparticles were prepared according to the previously published protocol [47]. Briefly, 300 mg of the corresponding polymer was dissolved in 4 mL of chloroform–acetone mixture (3:1 *v*/*v*) and dispersed into 80 mL of ice-cold water phase, containing 0.4 wt% of SDS at 1 wt% PVA, via simultaneous action of ultrasound homogenizer and magnetic stirrer at 750–900 rpm. Then, chloroform was evaporated, the particles were separated by centrifugation at 10,000× *g*, washed three times with distilled water and freeze-dried. 

#### 2.3.2. Supermacroporous Matrices Modification

##### Matrix Carboxylation

The 50 ± 10 mg piece of supermacroporous matrix was washed with water and then with 3 mL of 0.1 M NaOH for 20 min at 300 rpm shaking and room temperature. After that, the sample was washed three times with water. 

##### Matrix Amination

The piece was washed three times with 0.1 M MES buffer solution, pH 5.6 (MES-buffer). Then, 2 mL of HOBT 2 mg/mL solution in MES-buffer was added to the washed matrix, shaken at room temperature for 10 min, and cooled to 5 °C. A total of 1 mL of precooled EDC 2 mg/mL solution in MES-buffer was added to the mixture obtained and shaken at 300 rpm for 30 min at 0–5 °C. Further, the matrix was washed three times with water and three times with sodium borate buffer solution, pH 8.5 (BBS). A total of 2 mL of BBS, containing 10 µL of ethylenediamine (EDA), was added to the matrix and shaken at room temperature for 2.5 h. Then, the sample was washed several times with excess of water to remove the remaining EDA and vacuum-dried. In order to estimate the amino-groups content, the part of matrix was dissolved in chloroform and treated with TNBS. The concentration of bound TNBS was then measured at 335 nm with application of spectrophotometer and the previously plotted calibration curve. 

##### Kinetics of Chemisorption of Ox-PMAG on the Surface of Aminated Matrices (PLA-NH_2_ and PCL-NH_2_)

PLA-NH_2_ or PCL-NH_2_ matrix (50 ± 10 mg) was washed with BBS and placed into ox-PMAG 1 mg/mL solution in BBS (pH 8.5). The samples of supernatant solution were taken at specific time intervals and ox-PMAG concentration was estimated via the reaction with Schiff’s reagent [49] for polymeric aldehyde determination [43] with application of the previously plotted calibration curve at 550 nm. 

The amount of chemisorbed polymer (*Q_chemsorb_*) at different time intervals was calculated according to the following equation:

(1) $$Q_{chemsorb}=\;\frac{q_{chemsorb}}{m_{matrix}}=\frac{q_{0}-q_{i}}{m_{matrix}}=\frac{V_{0}C_{0}-V_{i}C_{i}}{m_{matrix}}$$

where *q_chemsorb_* is the amount of the polymer attached to the surface, which is equal to the loss of polymer quantity in the solution before (*q*_0_) and after (*q_i_*) adsorption; *V*_0_, *V_i_* and *C*_0_, *C_i_* are the volumes and the concentrations of the initial solution and the solution after chemisorption, respectively.

##### Isotherm of Chemisorption of Ox-PMAG on the Surface of PLA-NH_2_ and PCL-NH_2_ Materials

Several samples of PLA-NH_2_ or PCL-NH_2_ matrices (50 ± 10 mg each) were washed with BBS and placed into ox-PMAG solutions with different concentrations (from 0.1 to 5.0 mg/mL). After 16 h, the solutions were separated from the matrices and polymer concentration was estimated via the reaction with Schiff’s reagent. The amount of chemisorbed polymer (*Q_chemsorb_*) at different concentrations was calculated similarly to Equation (1).

##### Ox-PMAG-Protein Conjugates Synthesis

Firstly, the model protein, namely, chymotrypsinogen (CTRG) was labeled with Cy3-NHS. The 3-times molar excess of the label towards protein amount was used. The reaction was carried out in BBS in the dark at room temperature. After 1 h, the protein-Cy3 was concentrated with Vivaspin-column MWCO 10,000. Then, the conjugates of ox-PMAG with labeled proteins were obtained by mixing their solutions at 20-times molar excess of aldehyde groups towards the protein’s lysine ε-aminogroups. The conjugates were purified using centrifugal tube Vivaspin-columns with a membrane of MWCO 30,000.

##### Kinetics of CTRG-Cy3 Chemisorption on Polyester Matrix Surface Modified by Aldehyde Containing Ox-PMAG

The PLA or PCL matrix (50 ± 10 mg) with attached ox-PMAG was washed with BBS and placed into a solution of protein-Cy3 conjugate. At specific time intervals, the supernatant aliquots were taken and the absorbance of Cy3 at 555 nm was measured. The amount of chemisorbed conjugate (*Q_chemsorb_*) was calculated similarly to Equation (1).

##### Kinetics of Ox-PMAG-CTRG Conjugate Chemisorption on Amino-Polyester Matrix

The kinetic studies were performed similar to the procedure described for ox-PMAG chemisorption (see above). The detection of protein-ox-PMAG concentration in the supernatant was carried out via measurement of Cy3 absorbance at 555 nm. The amount of chemisorbed conjugate (*Q_chemsorb_*) was calculated similarly to Equation (1).

##### Isotherm of CTRG-Cy3 Chemisorption on the Polyester Matrix Surface Modified by Aldehyde Containing Ox-PMAG

Several samples of PLA-NH-PMAG-CHO or PCL-NH-PMAG-CHO matrices (50 ± 10 mg each) were washed with BBS and placed into CTRG-Cy3 solutions with different concentrations (from 0.01 to 2.0 mg/mL). After 4 h, the supernatant solutions were separated from the matrices and the absorbance of Cy3 was measured at 555 nm. The amount of chemisorbed labeled protein (*Q_chemsorb_*) was calculated similarly to Equation (1).

##### Determination of Unreacted Aldehyde Groups’ Quantity Remained on the Surface of PLA/PCL-NH-PMAG-CTRG Matrices

A number of modified matrices (see above) were placed into glycine (Gly) 1 mg/mL solution in BBS and left overnight at room temperature and at constant 300 rpm shaking. After 16 h, the supernatant solutions were separated from the matrices. They were three times washed with BBS to elutriate the residual amino acid. The Gly concentration in each solution was estimated using TNBS for the indication of amino groups. The absorbance of Gly-TNBS at 335 nm was measured. The molar quantity of residual aldehyde groups was calculated as following:

(2) $$q_{aldehyde}=\;\frac{C_{Gly\;initial}-\;C_{Gly\;after\;reaction}}{V\;MW_{Gly}},$$

*C_Gly before_* and *C_Gly after reaction_*—the concentration of glycine in a solution before and after the reaction with aldehyde groups on the surface (*C = D*_335_/*ε, D*—optical density, ε—coefficient obtained from calibration curve); *V*—solution volume (3 mL); *MW_Gly_*—molecular weight of glycine.

#### 2.3.3. Nanoparticles Modification

All stages described above for the matrices were very similarly applied for the nanoparticles (NPs) modification studies. The weight of NPs samples used at such experiments was 10 ± 2 mg. The 0.01 M NaOH solution was applied for carboxylation of the surface, because of the greater surface area of the particles. The buffer and reagents solutions exchange during NPs modification was performed via centrifugation at 11,000× *g* and further suspending of NPs in new solution. The purification of particles from unbound CTRG and ox-PMAG was performed with application of Vivaspin-columns with a membrane of MWCO 30,000.

### 2.4. Cell Culture Experiments

The preparation of matrices modified by poly-l-lysine (PLL), GRGDSP-peptide and BMP-2, as well as of NPs, modified by “self”-peptide and camel antibodies, was performed analogously to the procedures described above for the modification with model compounds. Typically, the 4 mL of 250 µg/mL solutions of the ligands in BBS were used to modify the 100 mg of the material.

#### 2.4.1. Cell Culture Experiments for Scaffolds Testing 

The AD44NN mesenchymal stem cells (MSC) non-commercial cell line derived from human adipose tissue was used in these studies. All cell culture experiments were conducted in accordance with European GLP standards for biological experiments. The sterilization of the solutions for materials modification and nutrition media was conducted via filtration through 0.22 µm membrane filters. The polymer matrices were sterilized by treatment with 70% isopropanol. The Minimum Essential Medium Eagle Alfa Modification, supplemented by 2.5% lysate of human platelet and antibiotics, was used for cell revitalization and cultivation. The cell cultivation was performed in a thermostated incubator (5% CO_2_, 37 °C).

##### Adhesion Experiment

Small pieces of modified PLA- or PCL-based matrices (3–4 mm in diameter) were placed into the wells of a 96-well plate, preliminary covered with agarose gel. The 10^4^ cells were added dropwise to each well. The cells were allowed to attach to the surface for 1 h in an incubator at 37 °C. Afterwards, the matrices were washed with fresh medium twice and MTT solution in basal medium was added. The samples were further incubated for 2 h at 37 °C and subjected to the standard MTT-test procedure [44]. 

The long-termed cell culture testing of modified PLA and PCL supermacroporous matrices was conducted at static and dynamic conditions. In both cases, 10^5^ cells were seeded onto the surface of the matrices.

##### Static Conditions

The matrices were incubated in 24-well plate, in the wells in which the medium was changed every 3 days. The cells were cultured for a week, after which the culture medium was replaced by an osteogenic differentiation medium in which the cells were cultured for additional 3 weeks.

##### Dynamic Conditions

The matrices were fixed in the bioreactor with a silicone ring in order to allow the culture medium to flow through the opened pores of the material. The cells were cultured for a week, after which the culture medium was replaced by an osteogenic differentiation medium in which the cells were cultured for additional 3 weeks. 

In order to analyze the results of long-term experiments, the cells on the matrices were stained by DAPI to visualize their amount according to the standard procedure [50]. In order to analyze the calcification of the matrices the staining with alizarin red [51] was performed.

#### 2.4.2. Ex Vivo Determination of Particles Phagocytosis by Peritoneal Macrophages

The 420 ± 70 nm particles were prepared and covalently modified by Cy3-NHS labeled BSA. One fraction of the particles was modified by “self-peptide” and another one, by small camel antibodies. Fifteen mice were separated into three groups (five animals in each): (1) Control group, treated with PLA-PMAG-BSA-Cy3 particles; (2) treated with PLA-PMAG-BSA-Cy3- “self-peptide” particles; (3) non-treated particles, correspondingly. A total of 1 mL of particle suspension in physiological solution (0.9% NaCl) with a concentration of 3 mg/mL was injected intraperitoneally. After 20 min, the animals were euthanized and 5 mL of physiological solution was introduced intraperitoneally to wash out the particles. From 2.5 to 3.0 mL of liquid was taken from peritoneum of each mouse. Then, 1 mL of retrieved liquid was placed on the coverslips located into the Petri dish and incubated for 1 h at 5% CO_2_ and 37 °C. Then, the dish content was washed with warm physiological solution and fixed with 4% paraformaldehyde solution for 40 min in the dark. The samples were then washed with physiological solution three times for 20 mins and stored in the freezer (4 °C) before measurement.

The samples obtained were stained in the dark with DAPI (0.36 µM in PBS) for 5 min at room temperature (25 °C) and washed twice with 0.01 M PBS, pH 7.4. The coverslips were attached to the microscope slides with DAKO mounting media (Agilent Technologies, Santa Clara, CA, USA). The cells were imaged with application of a confocal laser microscope Carl Zeiss «LSM 510 META» (Carl Zeiss, Basel, Germany) equipped with Plan-Apochromat 20x/0.8 M27 and C-Apochromat 40X/1.20 W CorrUV-VIS-IRM27 objectives. For excitation of Cy3 and DAPI fluorescence the 561 and 405 nm lasers were applied, correspondingly. The fluorescence detection was performed with >575 nm filter in the case of Cy3, and with a 420–480 nm filter for DAPI. The images in transmitted light were obtained by application of differential-interference contrast technique. The images were converted into JPEG by application of software supplemented to the confocal microscope. 

### 2.5. Statistical Analysis

The results were expressed as mean ± standard deviation (SD). A normality test and a test for equal variances were performed before running Student’s two-tailed t-test to compare results between two groups. In the case of unsatisfied correlation of the data, the normality Mann–Whitney-Rank-Sum test was performed. A *p* value < 0.05 was considered statistically significant.

## 3. Results and Discussion 

### 3.1. Materials Formation 

**Figure 1:**
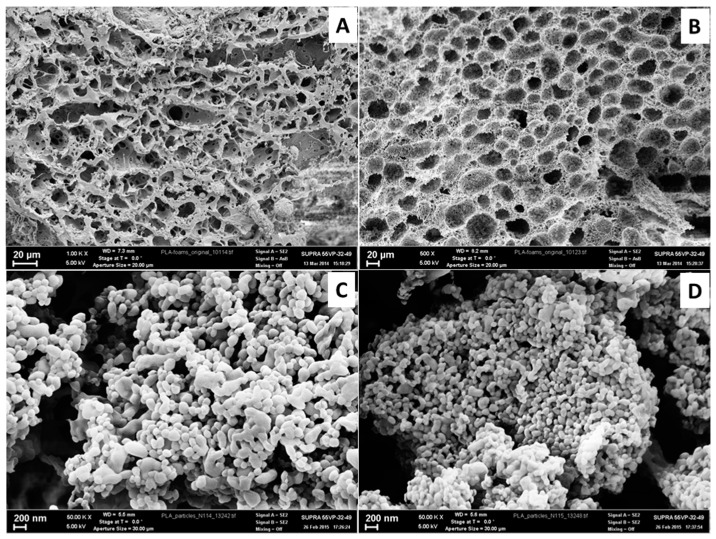
Morphology of polyester-based materials visualized by scanning electron microscope (SEM): (**A**) poly(lactic acid) (PLA) and (**B**) poly(ε-caprolactone) (PCL) supermacroporous matrix; (**C**) PLA and (**D**) PCL nanoparticles.

PLA- and PCL-based supermacroporous matrices were formed via thermally induced phase separation of their solutions in 1,4-dioxane/water mixture [46]. The PLA and PCL based nanoparticles were formed by single emulsion approach [47]. The SEM microphotographs of prepared materials are presented in Figure 1. One can observe that obtained matrices possessed large and interconnected pores that are necessary for 3D cell cultivation. The formed particles had a mean diameter of about 200 nm. Such particle size is appropriate for applications such as polymer particles in drug delivery, especially via intravenous injection.

### 3.2. Materials Modification

**Figure 2:**
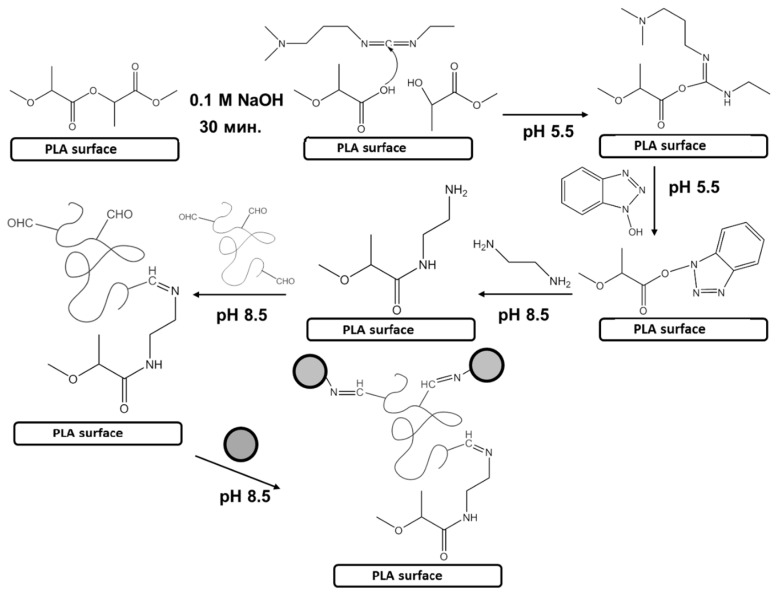
Scheme of biofunctionalization of polyester-based materials with application of oxidized poly(2-deoxy-*N*-methacriloylamido-d-glucose) (ox-PMAG).

**Figure 3:**
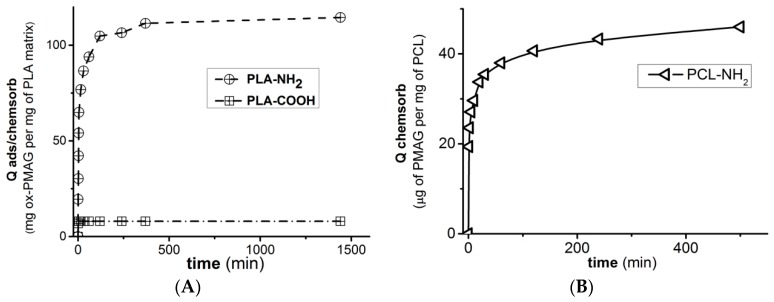
Kinetic curves of ox-PMAG adsorption: (**A**) Carboxylated and aminated PLA matrix surface, (**B**) aminated PCL matrix surface. Conditions: 0.01 M sodium borate buffer solution, pH 8.5, 25 °C, 300 rpm, [ox-PMAG] = 1 mg/mL.

The general chemical scheme of materials modification is presented in Figure 2. First of all, the material surface should be enriched with reactive carboxylic groups, which are performed by controllable treatment of the matrix with sodium hydroxide solution. The measured contact angle after the treatment of model PLA films showed that it was changed from 72° to 61°. Nevertheless, this hydrophilization did not allow for physical adsorption of ox-PMAG on the surface of a PLA-based supermacroporous matrix (Figure 3). Thus, the covalent interaction between the aldehyde-containing hydrophilic spacer and the surface is required. Initially, we have converted carboxyl groups into active esters and then introduced the amino groups to the surface by treatment with ethylene diamine. The surface concentration of carboxylic groups was determined via back titration of added sodium hydroxide excess with hydrochloric acid. The resulted amino groups were quantified by color reaction with TNBS (Table 1).

The higher concentrations of sodium hydroxide should be applied to the matrices as compared to the nanoparticles, due to the thick walls of polyester material in a slab shape. At the same time, a lower number of functional groups are obtained in this case because of the minor surface area of macroporous materials (Table 1). The nanoparticle surface is more easily activated by alkaline treatment, but one should be beware of the particle dissolution. We have observed 30% weight loss of the particle sample when 0.1 M NaOH was applied for 30 min. 

The difference in the amount of carboxylic groups formed on the surface of materials under study could be affected by two factors: Relative hydrophilicity of the surface (wettability) and surface area. The PLA is known to be more hydrophilic than PCL [52]. This explains the relatively greater number of carboxylic groups formed on the surface in the case of PLA nanoparticles rather than on the surface of PCL ones. The accessibility of the reaction surface area for such materials is close to each other. However, the surface area of supermacroporous materials seems to be different. This could be explained by different PLA and PCL crystallinity, which affects the formation of matrices more than the preparation of nanoparticles. The more crystalline PCL possess some amount of micropores in the walls of the macropores, and this increases the surface area and, consequently, the amount of formed carboxylic groups. 

The study of polyesters’ molecular weight change after treatment of materials with sodium hydroxide solution showed that the average molecular weight remained nearly the same both for the particles and matrices (*M_w_* (SEC, PLA) = 148,000; *M_w_* (SEC, PCL) = 114,000). At the same time, the molecular weight distribution growth was observed, due to the destruction of macromolecules on the surface. The *Ð* values for PLA and PCL nanoparticles were 2.21 and 2.08, while for PLA and PCL matrices such values constitute 1.93 and 1.75, correspondingly.

**Figure 4:**
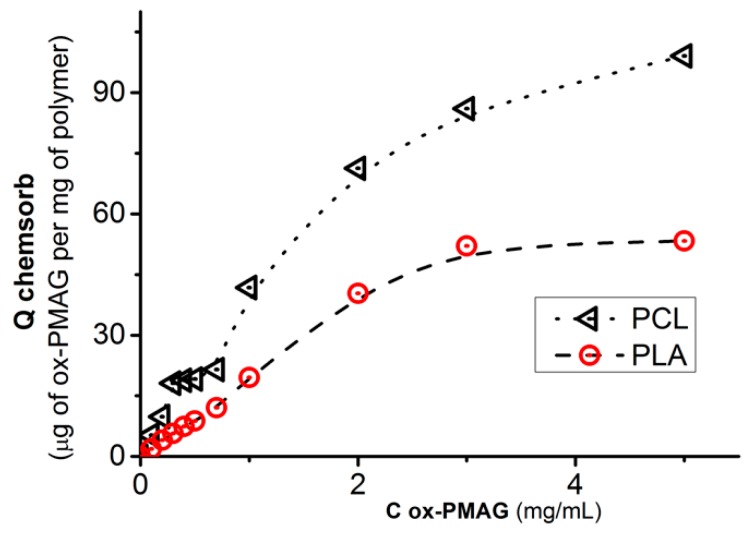
Isotherms of ox-PMAG adsorption on the surface of aminated PLA and PCL. Conditions: 0.01 M sodium borate buffer solution, pH 8.5, 25 °C, 300 rpm.

The chemisorption process of ox-PMAG on aminated surfaces (Figure 3) was quite fast and reached the plateau approximately within 1 h. Nevertheless, constructing the isotherms (Figure 4), in order to be sure that maximum polymer quantity was bound to the surface, we performed the reaction overnight. 

The ox-PMAG chemisorption isotherms demonstrate the high capacity of polyester surface towards aldehyde-bearing polymer (Figure 4). Surprisingly, the capacity of more hydrophobic PCL towards hydrophilic polyvinylsaccharide sorption was higher than that found for relatively hydrophilic PLA. This might be explained by a slightly higher number of amino groups introduced into the surface of PCL (see Table 1). 

**Figure 5:**
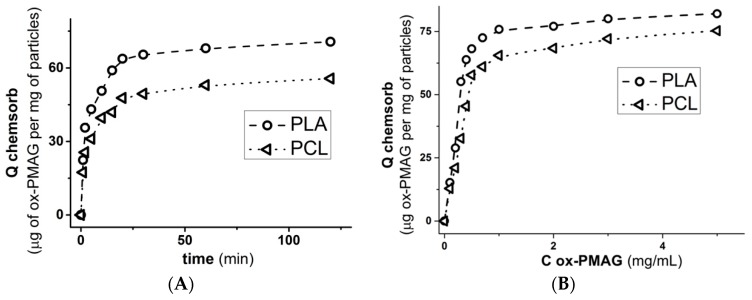
Kinetic curves (**A**) and isotherms (**B**) of ox-PMAG adsorption on the surface of aminated PLA and PCL particles. Conditions: 0.01 M sodium borate buffer solution, pH 8.5, 25 °C, 300 rpm.

The ox-PMAG adsorption on the surface of aminated PLA and PCL nanoparticles (Figure 5A) is more rapid than that on the matrices. This observation, as well as the shape of the isotherm (Figure 5B), could be explained by less pronounced diffusion limitations for ox-PMAG access to the amino groups on the surface of the particles in comparison to the matrices. Moreover, the exposure of amino groups for reaction with aldehyde-bearing polymer should be considered. This one will be greater in the case of nanoparticles due to higher surface/volume ratio in comparison with the matrices.

### 3.3. Biofunctionalization of Polyester Surfaces with Application of Ox-PMAG

#### 3.3.1. Supermacroporous Matrices

Two different strategies of aminated matrices biofunctionalization could be applied: (1) Chemisorption of ox-PMAG on the surface of aminated matrix and further attachment of biomolecules of interest; (2) modification of the ox-PMAG by bioligands followed by attachment of the resulted bioconjugate to the surface of polyester-based matrix using the residual aldehyde groups of the macromolecular spacer. 

**Figure 6:**
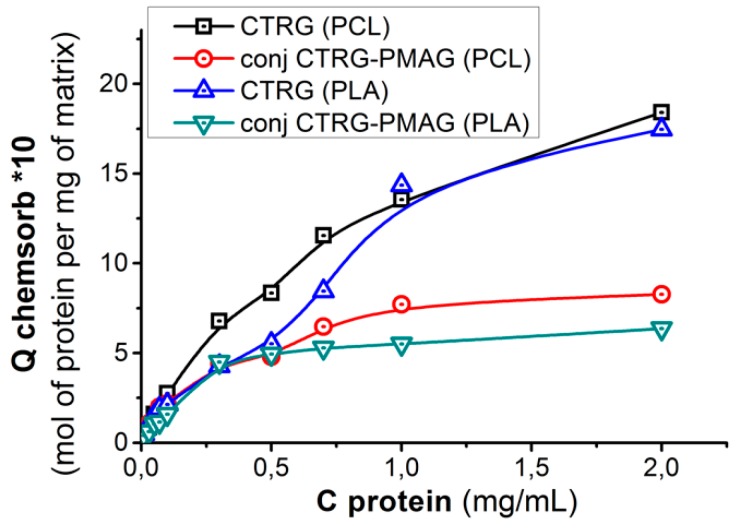
Isotherms of CTRG and PMAG-CTRG chemisorption on aminated PLA- and PCL-based surfaces. Conditions: 0.01 sodium M borate buffer solution, pH 8.5, 25 °C, 300 rpm.

For physico-chemical evaluation of peculiarities of surface modification we used a small stable protein, namely, chymotrypsinogen (CTRG). In order to compare the described approaches, we studied the chemisorption of CTRG on the surface modified with ox-PMAG and the chemisorption of ox-PMAG-CTRG conjugate (only the protein concentration was considered). The plotted isotherms (Figure 6) revealed the higher capacity of the matrix towards CTRG when the step-by-step approach was performed. This can be explained by the flattening of the hydrophilic spacer during its initial chemisorption that results in more accessible CHO-groups for the interaction with protein. In the case when conjugate formation was initially performed, the multivalent cross-linking of protein molecules might occur, resulting in compaction of PMAG macromolecules and participation of a higher number of aldehyde groups in the reaction of protein conjugation. Thus, in the latter case, a smaller number of functional groups stays for attachment to the surface. 

**Figure 7:**
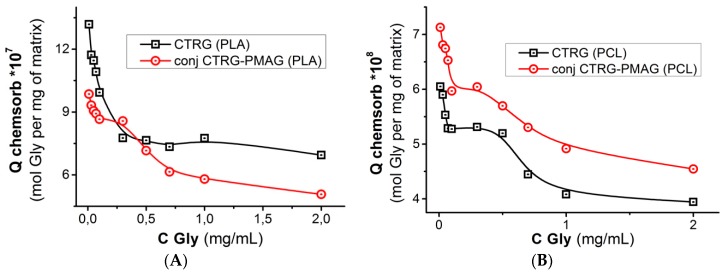
Dependences of Gly amount bound to the residual aldehyde groups of ox-PMAG on CTRG quantity immobilized on the surface: (**A**) PLA, (**B**) PCL. Conditions: 0.01 M sodium borate buffer solution, pH 8.5, 25 °C, 300 rpm.

To estimate the residual aldehyde group content for attachment of other biomolecules for multibiofunctional surface construction, we determined the amount of glycine (Gly) coupled to the surface, which was already modified by ox-PMAG and CTRG, or by PMAG-CTRG conjugate. The quantity of Gly coupled to the surface was plotted as a function of bound to the surface protein (Figure 7). Obviously, even at the very high surface quantity of bound protein the free aldehyde groups still exist and thus can couple the amino acid. 

#### 3.3.2. Nanoparticles

**Figure 8:**
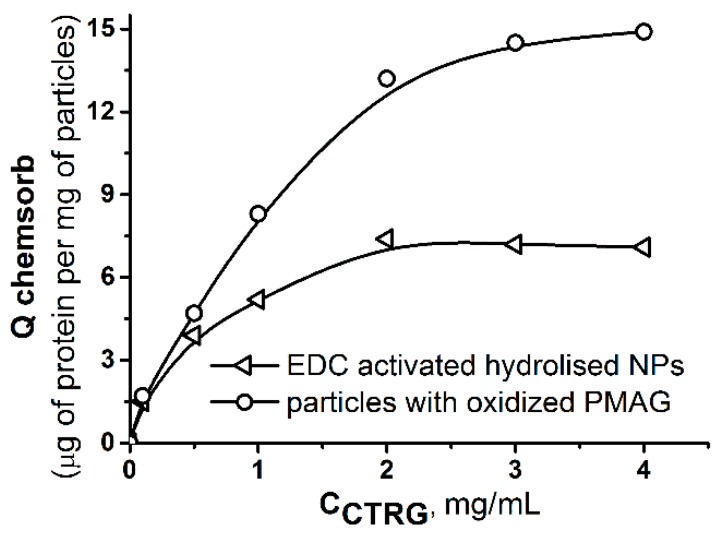
Isotherms bound for CTRG chemisorption on the surface of the PLA nanoparticles with activated carboxylic groups and covered with ox-PMAG. Conditions: 0.01 M sodium borate buffer solution, pH 8.5, 25 °C, 300 rpm.

In order to evaluate the effect of the nanoparticles covering with ox-PMAG on the quantity of protein bound to their surface, the isotherm of CTRG chemisorption was also plotted and compared to that obtained in the case of protein binding to carboxylated particles treated with EDC (Figure 8). 

One can observe that PLA nanoparticles covalently covered with ox-PMAG possess greater capacity towards CTRG. It means that a chosen spacer and functionalization approach allow multifunctional surface decoration to prolong particles circulation in a blood stream and to target their direction to specific cell types.

### 3.4. Study of the Effect of Bioligands Attachment on Cell Adhesion, Proliferation and Differentiation

#### 3.4.1. Cell Adhesion (Short-Term Cultivation)

**Figure 9:**
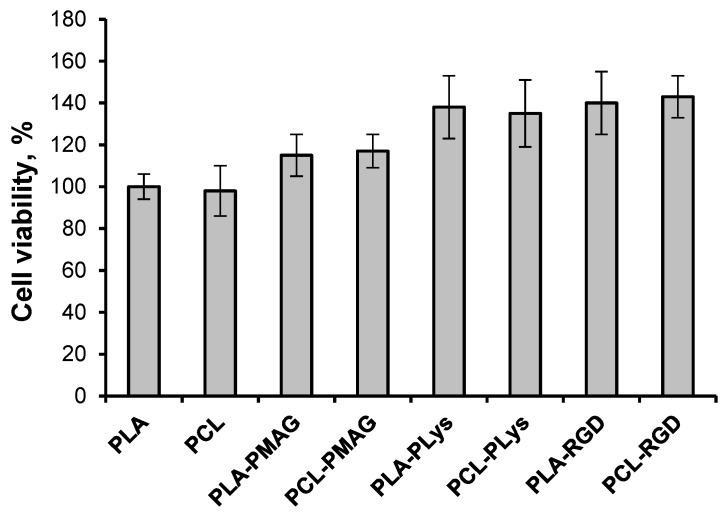
MTT-test results: cell incubation on initial PCL and PLA matrices and on those modified with ox-PMAG followed by poly-l-lysine (PLL) and RGD-peptide binding.

The successful application of polyesters as materials for tissue engineering requires providing the cell adhesion on the surface of such materials. In this regard, oligo- and polylysines and so-called RGD-peptides are of interest. The first type of molecules are known to enhance the electrostatic adhesion of cells to the surface of materials, while the second ones are responsible for specific cell adhesion via interaction with cells transmembrane receptors-integrins [22,45]. In order to obtain macroporous materials, which are able to attract the cells to their surface, the PLA and PCL matrices were modified by ox-PMAG according to the previously described strategy. Further, the poly-l-lysine (PLL) and GRGDSP peptide (RGD-peptide) were bound to the surface of the matrices. The adipose derived MSC were seeded on the scaffolds obtained and cell adhesion for 1 h was detected using the MTT-test (Figure 9). 

One can see (Figure 9) that cell viability on the modified materials did not decrease comparatively to that observed for non-modified polyester matrices applied as a control. On the contrary, the modification of matrices with PMAG led to a slight increase in the amount of viable cells on the material surface. This effect appeared to be more pronounced in the case of PLL and RGD-peptide coupling. Thus, the proposed method of biofunctionalization of polyester-based materials can be preliminarily considered as non-toxic for the cells and can be suggested for cell-instructive surfaces construction.

#### 3.4.2. Cell Proliferation and Differentiation (Long-Term Cultivation)

##### Cell Proliferation (Static Cultivation) 

**Figure 10:**
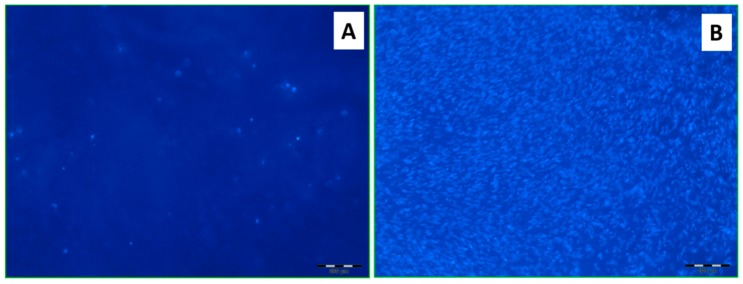
Fluorescent images of DAPI-stained mesenchymal stem cells (MSC) cells on the surface of the initial PLA-based matrix (**A**) and of that modified with RGD-peptide bound to ox-PMAG (**B**).

To study the efficiency of the developed modification approach, the matrices were modified with RGD-peptide via attachment to the ox-PMAG macromolecular spacer and incubated with MSC cells on a 24-well plate for one week in MEM, and for a further three weeks in osteogenic medium. Then, the cells adhered to the matrices were visualized by DAPI-staining. Obviously, the material modified using the suggested pathway was able to promote cells attachment and proliferation in a greater extent than non-modified PLA matrix (Figure 10).

##### Cell Differentiation (Dynamic Cultivation). 

**Figure 11:**
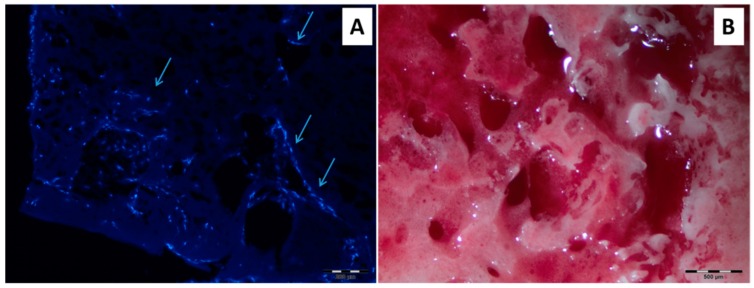
Results of one-month dynamic cultivation of MSC on modified PLA materials: (**A**) DAPI-stained slice (cells ingrowth into the material is marked with arrows), (**B**) matrix was stained with alizarin red (red color detects the calcification process).

The matrices modified with RGD-peptide were subjected to one-month cultivation in a flow-through bioreactor to explore the cell differentiation process. As in the case of static cultivation, in the first week the matrices with cells were incubated in MEM and for a further three weeks in osteogenic medium. The matrices after long-term cultivation were sliced and analyzed by DAPI-staining of adhered cells and detection of calcium via staining with alizarin red (Figure 11A,B). The presented results demonstrated the cells ingrowth into the scaffolding material and further mineralization (calcification) that was not observed for non-modified PLA material. Thus, the prepared biomimetic supermacroporous matrices can be considered as perspective scaffolds for bone tissue engineering. 

### 3.5. Phagocytosis of Nanoparticles

The effect of surface composition on the biological fate of the particles represents quite an important area of research. Being the foreign object for the organism, such particles are subjected to the interaction with immune cells.

The attachment of antibodies to the surface of the particles is sometimes used for active targeting of such systems to the diseased tissues [53]. The small camel IgGs are of particular interest in this regard [54]. Nevertheless, such modification could possibly increase the phagocytosis of particles due to opsonization effect. In order to prevent fast capturing of particles after injection into organism by phagocytes, the surface modification by PEG is often used [55]. Quite recently the new biomolecule —so-called “self-peptide”—was proposed for control of phagocytosis [48]. To show the possibility of particles modification with ox-PMAG to control phagocytosis, PLA-ox-PMAG nanoparticles were modified with BSA-Cy3, BSA-Cy3-“self-peptide” and camel antibodies labeled with Cy3. BSA was used as a protein carrier for the peptide to unify the surface of particles for correct comparison of particles’ biological properties.

The PLA nanoparticles prepared in this study possess a diameter of 420 ± 70 nm. The particles of such size are known to be well recognized and phagocyted by macrophages even without any modification [56]. However, the effect of such particles’ surface composition on the phagocytosis was studied.

**Figure 12:**
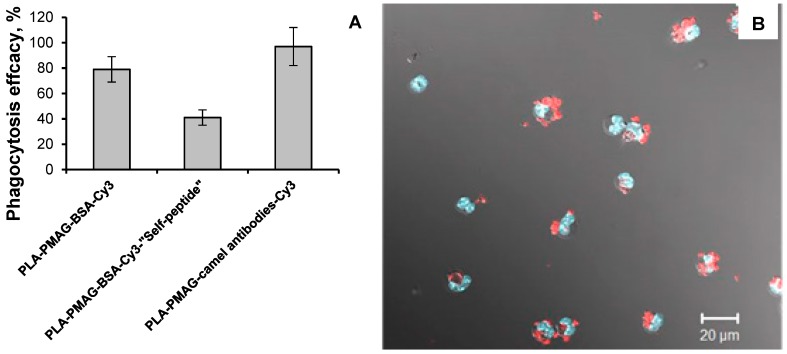
Results of phagocytosis of functionalized PLA nanoparticles: (**A**) Phagocytosis efficacy found for particles modified with different biomolecules; (**B**) image illustrating the phagocytosis process.

The nanoparticles were injected into the peritoneal cavity of mice and then the macrophages were extracted for further phagocytosis study. Phagocytosis efficiency was evaluated by calculation of macrophages with particles inside and referring quantity obtained for the entire amount of macrophages. Cy3 label was used to recognize the particles aggregates inside DAPI-stained macrophages. The results obtained are presented in Figure 12. 

The results of completed experiments showed the suitability of such modification to control the phagocytosis of particles. Interestingly, that modification of particles by polyclonal camel IgG has slightly increased the particles’ phagocytosis efficacy. This could be explained by the abovementioned opsonization effect, which makes the particles more recognizable for the macrophages. The increasing of particles’ phagocytosis could be very useful in the strategies of “cell-mediated drug delivery” [57]. In such strategies the cells are utilized as drug delivery vehicles. The immune cells are quite interesting candidates as they could migrate to the inflammation sites via chemotaxis-driven pathways. Thus, the effective phagocytosis of particles with an encapsulated drug will lead to effective drug loading inside the cells.

On the opposite side, the modification of the particles surface by “self-peptide” provided the substantial decrease of particles phagocytosis efficacy. For this reason, such a type of modification seems to be quite useful for preparation of particles, which are intended for intravenous injection and prolonged circulation into the bloodstream. 

## 4. Conclusions

The simple approach to biofunctionalization of polyester-based materials was proposed and realized for construction of scaffolds for bone tissue engineering, as well as for nanoparticles suitable for drug delivery. It was shown that the developed approach allows for increasing the capacity of the surface towards the coupling of different biospecific ligands. The coupling of several biomolecules of different origins was shown to be possible. Thus, the proposed method allows the formation of multibiofunctional surfaces. Additionally, it allows the surface hydrophilization and distancing of the attached molecules from the polyester hydrophobic surface that is important for the preservation of bioligand activity. Moreover, the saccharide residues presented in glycopolymer spacer (PMAG) can also play a role in the “glyco-related biointeractions”.

The proposed biofunctionalization approach was found to be applicable for the preparation of biomimetic materials. In particular, the biofunctionalized supermacroporous matrices were suitable for inducing adipose derived MSCs adhesion and differentiation into osteoblasts, but nanoparticles modified with bioligands were able to control the phagocytosis.

## Figures and Tables

**Table 1:** The effect of sodium hydroxide concentration on the amount of introduced COOH- and followed NH_2_-groups in the PLA/PCL-based matrices and particles.

Polyester	[NaOH], M	Sample Weight, mg	[COOH], nmol/mg (Titration)	[NH_2_], nmol/mg (Photometrical)
Matrices				
PLA	0.1	52	4.3 ± 0.2	2.2 ± 0.1
	1.0	47	5.1 ± 0.3	4.5 ± 0.2
PCL	0.1	51	6.8 ± 0.4	7.2 ± 0.4
	1.0	55	8.8 ± 0.5	8.1 ± 0.4
Particles				
PLA	0.01	12	8.4 ± 0.4	6.5 ± 0.3
	0.10	15	14.1 ± 0.7	11.0 ± 0.6
PCL	0.01	10	6.0 ± 0.3	3.9 ± 0.2
	0.10	13	10.3 ± 0.5	7.8 ± 0.4
